# Suppressing Li Dendrites via Electrolyte Engineering by Crown Ethers for Lithium Metal Batteries

**DOI:** 10.1007/s40820-020-00501-6

**Published:** 2020-08-07

**Authors:** Shanqing Zhang

**Affiliations:** Centre for Clean Environment and Energy, School of Environment and Science, Gold Coast Campus, Griffith University, Southport, QLD 4222 Australia

**Keywords:** Li dendrites, Crown ethers, Lithium metal batteries, Electrolyte

## Abstract

Electrolyte engineering is considered as an effective strategy to establish stable solid electrolyte interface (SEI), and thus to suppress the growth of lithium dendrites. In a recent study reported in Advanced Functional Materials by Ma group, discovered that strong coordination force could be founded between 15-Crown-5 ether (15-C-5) and Li+, which facilitates the crown ether (15-C-1) to participate in the solvation structure of Li+ in the electrolyte for the same purpose. Such a novel strategy might impact the design of high-performance and safe lithium metal batteries (LMBs).

Electrolyte engineering is considered as an effective strategy to establish stable solid electrolyte interphase (SEI) and thus to suppress the growth of lithium dendrites. A recent study reported in *Advanced Functional Materials* by Ma’ group, discovered that strong coordination force could be founded between 15-Crown-5 ether (15-C-5) and Li^+^, which facilitate the crown ether (15-C-1) to participate in the solvation structure of Li^+^ in the electrolyte for the same purpose. Such a novel strategy might impact the design of high-performance and safe lithium metal batteries (LMBs) [1].

LMBs have attracted escalating attention due to the ultrahigh theoretical capacity, which is ten times of lithium-ion batteries (LIBs). However, the potential safety issue of LMBs is throttling the application due to the significant volume change and uncontrolled dendrites growth of lithium (Li) dendrites [2–4]. Due to the super reactivity, the parasitic reaction between Li metal and electrolyte will react quickly to form the SEI film. However, the fragile and uneven SEI film is easily broken, leading to malignant dendritic growth and electrolyte consumption. To solve this problem, some functional additives that could coordinate with Li^+^ are introduced in liquid electrolytes, which are beneficial to the construction of stable SEI film [5]. The competitive combination behavior between the electrolytes and the additives with Li^+^ will determine the migration and deposition of Li^+^ or Li^+^ clusters [6].

In Ma’s work in *Advanced Functional Materials*, the mechanism of the strong coordination between 15-Crown-5 ether (15-C-5) and Li^+^ is used to inhibit the uneven growth of Li dendrites as illustrated in Fig. 1 [1]. As demonstrated by the charge density simulation, the Li^+^ lies in the center of 12-C-4 and 15-C-5 to form four or five Li–O bonds, respectively (Fig. 2a, b), but Li^+^ lies on one side of 18-C-6 and to form only four Li–O bonds (Fig. 2c). So, 15-C-5 possessed the strongest combination ability to participate in the solvation of Li^+^. Because of this, the Li^+^/15-C-5 complex could aggregate and distribute evenly at the Li metal surface due to the electrostatic adsorption, building up a protective layer to reduce the contact between electrolyte solvent and Li metal. Fortunately, the LiF is not affected in this process; in fact, LiF is the highest content component in SEI film (Fig. 2d). In addition, due to the existence of Li^+^/15-C-5 protective layer, Li^+^ is distributed uniformly on the Li metal surface, which facilitates the formation of the smooth and dense SEI film and drives uniform Li deposition (Fig. 2e). As a result, the crown ether15-C-5 is beneficial to the long-term performance of Li symmetric battery with 170 cycles, which is twice of that in the blank electrolyte (Fig. 2f). The most significant contribution of this work by Ma group is that it theoretically and experimentally demonstrates the proposed electrolyte additive helps the formation of superior SEI film through taking part in the processes of the solvation of Li^+^, the nucleation and growth of Li dendrites.

**Fig. 1:**
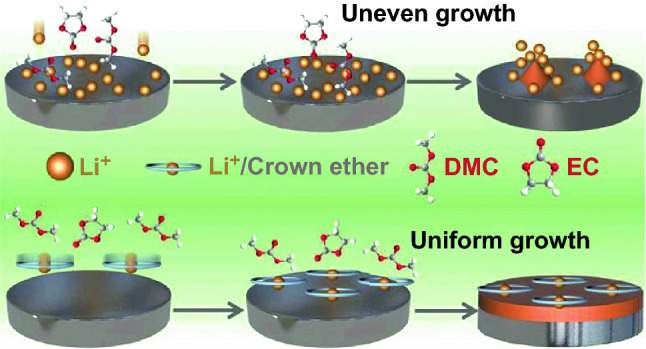
Illustrative schemes of the growth of Li dendrites in **a** black electrolyte and **b** crown ether-contained electrolyte. Adapted with permission from Ref. [1]

**Fig. 2:**
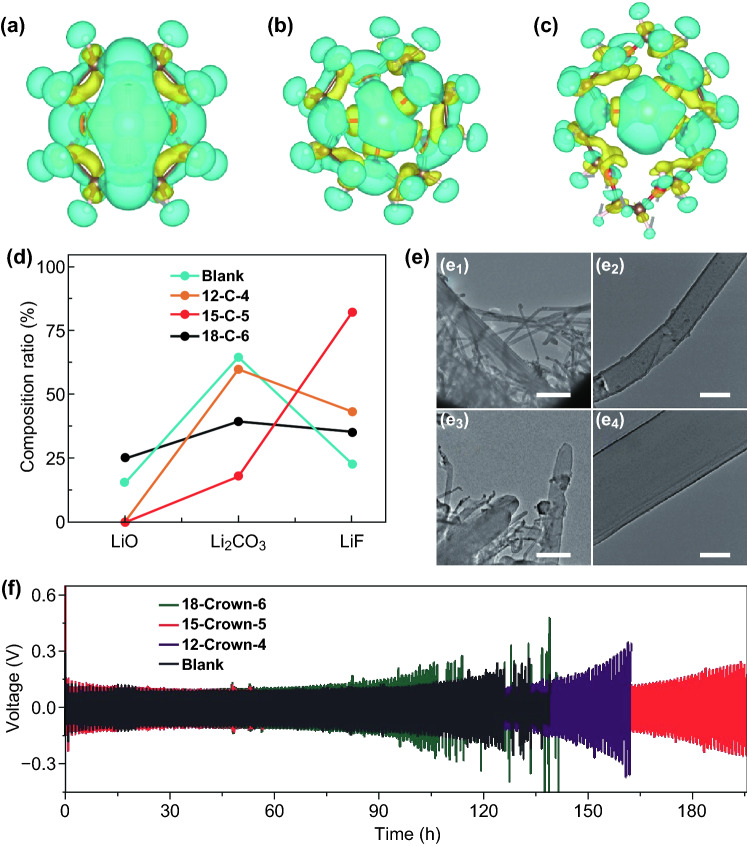
Charge density simulation of **a** Li^+^/12-C-4, **b** Li^+^/15-C-5, and **c** Li^+^/18-C-6. **d** The percent of the different compositions comes from Li 1 s XPS spectra. **e** The cryo-electron microscopy (cryo-EM) of metal dendrites in blank electrolyte (e_1_&e_2_) and 2.0 wt% 15-C-5 contained electrolyte (e_3_&e_4_) (scale bars: 2 µm and 200 nm for e_1_&e_3_ and e_2_&e_4_, respectively). **f** Electrochemical behavior of symmetric cell in the blank electrolyte and 2.0 wt% 12-crown-4 (12-C-4), 2.0 wt% 15-C-5, 18-crown-6 (18-C-6) contained electrolyte. Adapted with permission from Ref. [1]

In summary, Ma and coworkers demonstrate that electrolyte engineering is an effective strategy to establish SEI film to suppress dendrite growth in LMBs, and the crown ether 15-C-5 is a novel and promising electrolyte additive for high-performance and safe LMBs.
